# Spin density wave instability in a ferromagnet

**DOI:** 10.1038/s41598-018-23555-4

**Published:** 2018-03-27

**Authors:** Yan Wu, Zhenhua Ning, Huibo Cao, Guixin Cao, Katherine A. Benavides, S. Karna, Gregory T. McCandless, R. Jin, Julia Y. Chan, W. A. Shelton, J. F. DiTusa

**Affiliations:** 1 Department of Physics and Astronomy, Louisiana State University, Baton Rouge, LA 70803 USA; 2 Quantum Condensed Matter Division, Oak Ridge National Laboratory, Oak Ridge, TN 37831 USA; 3 Department of Chemistry and Biochemistry, The University of Texas at Dallas, Richardson, TX 75080 USA; 4 Department of Chemical Engineering, Louisiana State University, Baton Rouge, LA 70803 USA

## Abstract

Due to its cooperative nature, magnetic ordering involves a complex interplay between spin, charge, and lattice degrees of freedom, which can lead to strong competition between magnetic states. Binary Fe_3_Ga_4_ is one such material that exhibits competing orders having a ferromagnetic (FM) ground state, an antiferromagnetic (AFM) behavior at intermediate temperatures, and a conspicuous re-entrance of the FM state at high temperature. Through a combination of neutron diffraction experiments and simulations, we have discovered that the AFM state is an incommensurate spin-density wave (ISDW) ordering generated by nesting in the spin polarized Fermi surface. These two magnetic states, FM and ISDW, are seldom observed in the same material without application of a polarizing magnetic field. To date, this unusual mechanism has never been observed and its elemental origins could have far reaching implications in many other magnetic systems that contain strong competition between these types of magnetic order. Furthermore, the competition between magnetic states results in a susceptibility to external perturbations allowing the magnetic transitions in Fe_3_Ga_4_ to be controlled via temperature, magnetic field, disorder, and pressure. Thus, Fe_3_Ga_4_ has potential for application in novel magnetic memory devices, such as the magnetic components of tunneling magnetoresistance spintronics devices.

## Introduction

Incommensurate spin-density wave (ISDW) phases have long been known to be generated from paramagnetic (PM) metals either through a Fermi surface (FS) instability known as nesting or through the coupling of local magnetic moments via the Ruderman-Kittel-Kasuya-Yosida (RKKY) interaction^1,2^. Interestingly, no ISDW phase stemming from an instability in the spin polarized FS of a FM metal has been previously identified. We have discovered such a material in the transition metal binary compound Fe_3_Ga_4_ that hosts both FM and ISDW phases at zero applied field. Here, both of these phases are stable over wide temperature (*T*) ranges with the FM phase evident at temperatures above and below the *T* range of stability of the ISDW phase. Furthermore, we have discovered the mechanism for the formation of the ISDW state in Fe_3_Ga_4_, which is distinct from any previously identified and that makes clear the connection between the FM and ISDW states. This mechanism, indicated by our data and simulations, is likely not limited to Fe_3_Ga_4_, suggesting that ISDW order or spin fluctuations stemming from spin-polarized FS instabilities should be investigated in a wide range of complex magnetic materials.

ISDW phases caused by FS instabilities are common in *d*-electron systems such as the prototypical ISDW system Cr^3,4^, while the local moment picture is usually associated with magnetism in rare earth materials where the magnetic moments derive from well localized *f*-electrons^5–9^. ISDW phases are commonly associated with antiferromagnetic (AFM) interactions, as opposed to incommensurate helical states which are more aptly described as being derived from FM states. In fact, transitions between ISDW and FM states in zero applied field are extraordinarily rare in contrast to helical magnets where transitions to FM ground states are more common^10,11^. There are only a handful of FM materials that display ISDW phases over narrow *T* ranges with distinct, fairly restrictive mechanisms. These include: ferromagnets which seemingly avoid quantum criticality via a transition to a small ordered moment ISDW, Nb_1−*y*_Fe_2+*y*_, YbRh_2_Si_2_, and PrPtAl^12–15^; a frustrated magnetic insulator described as a Kagomé staircase system that undergoes transitions between several magnetic states, Co_3_V_2_O_8_^16^; and a very small number of Ce and U compounds which display ISDW states over a very narrow *T* range just above their FM transitions and where the mechanism is unclear, CeRu_2_Al_2_B and UCu_2_Si_2_^17–19^. The paucity of examples that we are able to find in the literature stemming from decades of intense investigation of magnetic materials is surprising.

In this article, we focus on the investigation of the magnetic and electronic structure of Fe_3_Ga_4_ because previous magnetization, *M*, measurements indicated that there may be an interesting and unusual re-entrant FM phase surrounding an AFM phase at both low and high *T*^20^. Neither the magnetic structure, nor the character of the magnetic phases had been identified. The transitions between FM and AFM phases in Fe_3_Ga_4_ can be tuned via *T*, magnetic field, and pressure^20–23^. We discover through neutron scattering measurements that Fe_3_Ga_4_ adopts an ISDW ordering between *T*_1_ = 60 and *T*_2_ = 360 K where the magnetic moments are oriented nearly transverse to the incommensurate wavevector. This magnetic structure is locally FM with neighboring magnetic moments being aligned but having a sinusoidal amplitude modulation occurring over several crystalline unit cells. In addition, we confirm that this material appears to display a thermodynamically odd re-entrant ferromagnetism above *T*_2_^20^. A clue as to the cause for this unusual behavior comes from simulations of the electronic structure. In the non-magnetic case, our calculations do not indicate regions of FS nesting. However, we discovered significant nesting in the FM majority-spin FS with a nesting vector that is in excellent agreement with the measured ISDW wavevector, *q* = (0 0 0.29). The FS instability along with anomalies in the charge transport support an itinerant mechanism for the magnetism in Fe_3_Ga_4_. This indicates that the ISDW emerges from an unstable FS in the *FM phase* so that a return to ferromagnetism is required prior to reaching the high *T* PM phase above *T*_3_ = 420 K. This mechanism, a FS instability in the majority band of a ferromagnet that drives a transition to an ISDW, has, to our knowledge, not been previously considered.

## Results

**Figure 1:**
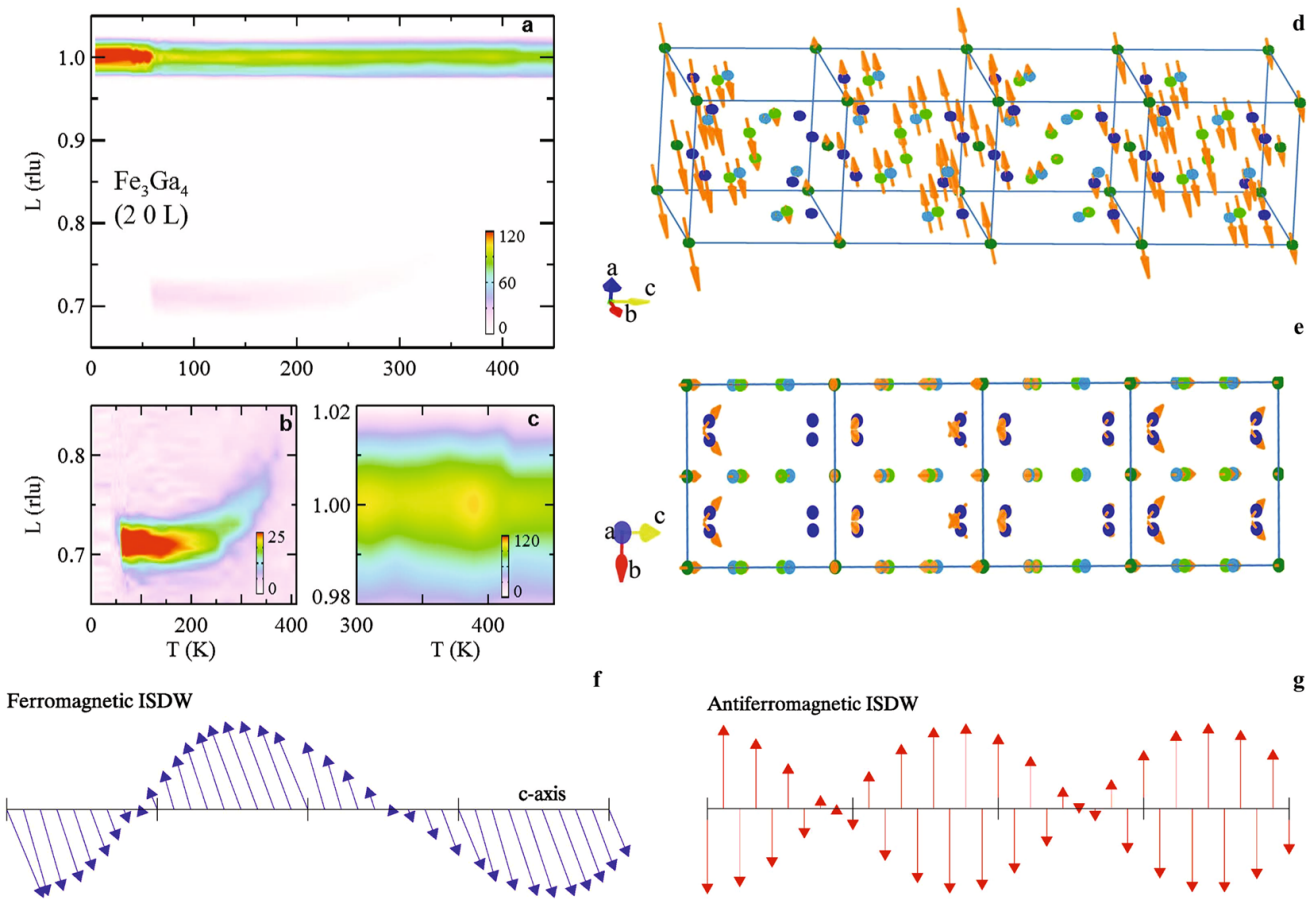
Neutron Diffraction. Temperature, *T*, dependence of the neutron scattering intensity along the (2 0 *L*) direction. Scans were performed in increments in *L* of 0.005 reciprocal lattice units (rlu) for $5\le T\le 450$ K in increments of 5 K. Color-bars indicate the scattering intensity in counts/s. Intensity plots displaying scattering (**a**) over the full *q* and *T* range of the data, (**b**) near the incommensurate wave-vector (2 0 1-*δ*) for $5\le T\le 450$ K, and (**c**) in proximity to (2 0 1) for $300\le T\le 450$ K. (**d**) Magnetic structure at 100 K over a four unit cell length along the *c*-axis depicting the ISDW state. Solid circles represent the 4 different Fe sites within the Fe_3_Ga_4_ unit cell. Structural data can be found in Supplementary Table S1. (**e**) Magnetic structure at 100 K viewed along the *a*-axis highlighting the non-collinear magnetic structure determined from the refinement of the neutron diffraction data. (**f**) Schematic of a modulated ferromagnetic ordering similar to the ISDW state in Fe_3_Ga_4_. The lattice constant is indicated by the tick marks and the tilting of the magnetic moments along the modulation direction (*c*-axis) is representative of the ISDW state determined for Fe_3_Ga_4_. (**g**) Schematic of the more common ISDW state found in antiferromagnets such as Cr.

**Figure 2:**
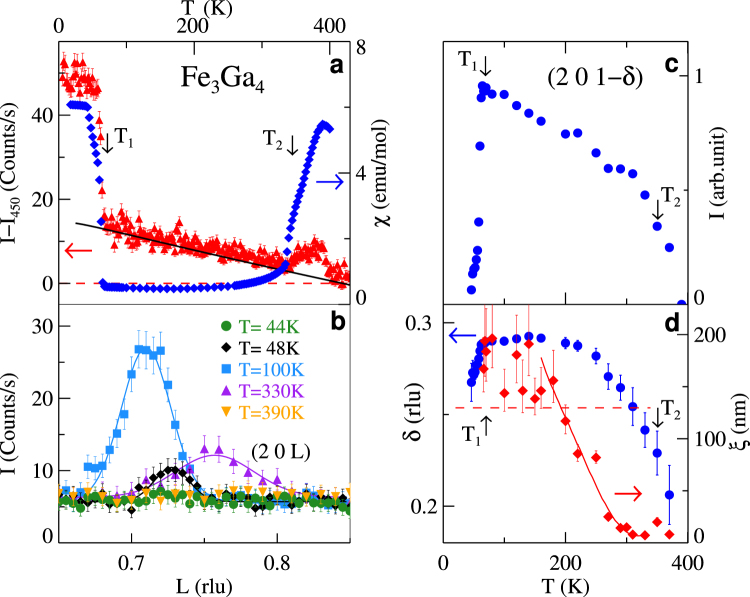
Temperature dependent magnetic scattering and magnetic susceptibility. (**a**) Neutron scattering intensity at the (201) Bragg peak position, *I* − *I*_450_, (red triangles) and magnetic susceptibility, *χ*, at 100 Oe (blue diamonds) vs. *T*. *I* − *I*_450_ is the intensity, *I*, measured after subtraction of the intensity at 450 K, *I*_450_. Solid line is a fit of the Debye model^43^ to the data between 90 and 300 K with Θ_*D*_ = 125 K^20^. (**b**) Scattering along the (2 0 *L*) direction at several representative temperatures demonstrating the incommensurate order. (**c**) Integrated intensity of the scattering at (2 0 1−*δ*) vs. *T*. (**d**) *T* dependence of *δ* (blue bullets) and the correlation length of the incommensurate scattering, *ξ*, (red diamonds) vs. *T*. Solid line is a guide to the eye. Dashed line is the instrumental resolution. Transition temperatures *T*_1_ and *T*_2_ are indicated in frames a, c, and d.

Fe_3_Ga_4_ forms in a monoclinic (*C*2/*m*) crystal structure with no evidence of a structural change associated with the magnetic phase transitions^20,22–25^. The description of the crystal structure is available in the Supplementary Information. A summary of our neutron scattering data is presented in Fig. 1a, which displays the intensity map for the scattering in the (2 0 *L*) reciprocal lattice direction. Three features of this plot represent our main experimental findings. First, the large increase in the scattering cross section at the (2 0 1) Bragg peak position below ~60 K (*T*_1_) demonstrates FM ordering consistent with the magnetic susceptibility, *χ*, (Fig. 2a) and previous measurements of *M*^20,22,23,26,27^. Second, for *T* just above *T*_1_, the reduced scattering at the Bragg peak position is accompanied by an increase in scattering centered at (2 0 0.71) indicating the existence of an incommensurate magnetic phase. The *T* and *q* dependence of this scattering contribution can be better viewed in Fig. 1b and in Fig. 2b,c where an abrupt loss of scattering is evident below *T*_1_ while a more continuous change is seen above 300 K. At these higher temperatures, the scattering moves to somewhat larger *q* before this signal is reduced below the background at *T* > 360 K (*T*_2_). Third, in this same *T* range, the scattering at the (2 0 1) Bragg peak position increases (Figs 1a,c and 2a) before decreasing at *T* > 420 K. This is consistent with the paramagnetism found in *χ* and *M* above *T*_3_ = 420 K^20^. Thus, our data indicate a FM ordered state below *T*_1_ transitioning to an incommensurate magnetic state for *T*_1_ < *T* < *T*_2_ along with a re-emergence of ferromagnetism between *T*_2_ and *T*_3_.

Surveys of a large number of reciprocal lattice positions have allowed a full refinement of the magnetic structure. The details of the refinement are presented in the Supplementary Information along with the results presented in Supplementary Table S2 for the low *T* FM state (*T* < *T*_1_) and Supplementary Table S3 for the incommensurate magnetic order at 100 K. In Supplementary Table S2, we report the size of the magnetic moment on each of the Fe sites refined with the magnetic moment constrained to lie along the crystallographic *c*-axis. These are in good agreement with the average magnetic moment determined from *M*^20,23,26^. When the constraint is removed a small contribution along the *a* and *b* directions is found that is smaller than the error in the refinement (~0.3 *μ*_*B*_). In Fig. 2a we present a comparison of the scattering intensity, after subtraction of the Bragg scattering in the paramagnetic state at 450 K, to *χ*, measured on the same crystal. The two regions in *T* where *χ* is large correspond well with the increased scattering at (201).

**Figure 3:**
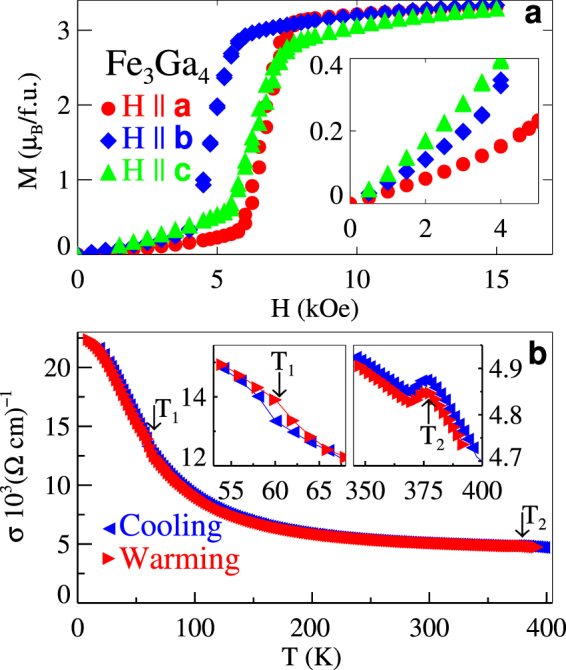
Magnetization Anisotropy and Electrical Conductivity. (**a**) Magnetization, *M*, vs. magnetic field, *H*, along the 3 principle crystal axes, *a*, *b*, and *c*, within the incommensurate spin density wave phase at 100 K. (**b**) Temperature, *T*, dependence of the electrical conductivity conductivity, *σ*, measured with current along the *c*-axis. Insets: changes to *σ* at the magnetic phase transitions. Transition temperatures *T*_1_ and *T*_2_ are indicated.

The result of the refinement of the magnetic scattering at 100 K (incommensurate phase) is significantly more complex with the best fit to our data consisting of a structure that closely resembles that of an ISDW having a propagation vector of *q* = (0 0 0.29). After establishing the ISDW character of the magnetic state, we have further constrained the model such that crystallographically equivalent sites were required to have the same magnetic moment amplitude. The results of this refinement are displayed in Fig. 1d,e. In Supplementary Table S3 the magnitude of the magnetic moments in one particular unit cell is presented, but we note that the incommensurate nature of the ordering results in moments that vary from cell to cell accordingly. The refinement places the magnetic moments mostly along the *a*-axis with a maximum magnitude of 2.31(6) *μ*_*B*_. However, there is also a considerable contribution along the *c*-axis of amplitude of $\mathrm{0.40(1)}{\mu }_{B}\le {m}_{max}\le \mathrm{0.58(6)}\,{\mu }_{B}$ as well as along the *b*-axis for one of the four crystallographically unique Fe sites (see Supplementary Table S3). Thus, there is a significant non-collinear and non-coplanar magnetic moment indicated by our data. Previously, we had speculated about such a non-coplanar magnetic moment based upon a large topological Hall effect in the range of temperatures and fields where the AFM is stable^20^ and the data presented here confirm this interpretation. The ISDW magnetic structure indicated by our refinement of the neutron diffraction data is consistent with the anisotropy in *M* presented in Fig. 3a. Here, significant differences in *M*, including the field induced metamagnetic transition, measured with fields along the 3 principle crystal axes rule out any simple helimagnetic structures.

The magnetic state at 100 K represented in Fig. 1d retains a FM orientation of neighboring magnetic moments with an amplitude that is modulated by the wave vector *δ* = 0.29 in reciprocal lattice units. This is distinct from the simpler ISDW materials, such as Cr^3^, where neighboring sites are antiferromagnetically aligned. Thus, the ISDW state appears to be a long wavelength modulation of a FM state as demonstrated schematically in Fig. 1f and compared to the ISDW state associated with antiferromagnetism (Fig. 1g). The average size of the magnetic moments in the ISDW state are within error of those in the FM state indicating a conservation of total moment magnitude at *T*_1_. However, there is a significant change in the direction of the magnetic moments in the FM and ISDW structures. The complexity of the ISDW phase and the significant differences in the two competing magnetic phases highlight the question of the mechanism driving the ISDW ordering and its instability at low *T*.

The evolution of *δ* and the correlation length for the magnetic scattering, *ξ*, with *T* can lend insight into the character of the magnetic state and the competing interactions which are of clear importance in Fe_3_Ga_4_. In Figs 1b and 2b,d, the *T* evolution of *δ* is displayed. This *T* dependence is usually associated with the competition between the electronic degrees of freedom responsible for the incommensurate nature of the wavevector and the lattice degrees of freedom which prefer a commensurate density wave^28^. Here, we observe a decrease in *δ* as the FM phases are approached either by warming or cooling with a large decrease in *δ* apparent above 250 K. Preceding this decrease in *δ* is a precipitous reduction in *ξ* as determined from the widths of the (2 0 1−*δ*) scattering peak for *T* > 200 K (Fig. 2b,d). Above 300 K, *ξ* has decreased such that it is equivalent to several wavelengths of the ISDW phase (2*π*/*δ*). Thus, there is an extended *T* range where only short range ordering exists.

Independent of the mechanism responsible, most ISDW phases are accompanied by a discontinuous decrease in conductivity, *σ*, and an increase in the Hall constant as the result of a partial FS gapping. This is a consequence of either FS nesting^2^ or simply the additional periodicity associated with the ISDW phase. Fe_3_Ga_4_ does not disappoint in this regard as we identify both a decreased *σ*(*T*) at *T*_1_ and a somewhat smaller increase at *T*_2_ (Fig. 3b) indicating a significant change to the FS^20,23^. Note that there is some sample-to-sample variation in *T*_2_ for our crystals likely caused by small differences in the density of antisite defects^20^ so that *T*_2_ = 375 K in Fig. 3b. Furthermore, the ordinary Hall coefficient is observed to undergo large changes at both *T*_1_ and *T*_2_^20^ also consistent with a significant change to the FS at these temperatures.

**Figure 4:**
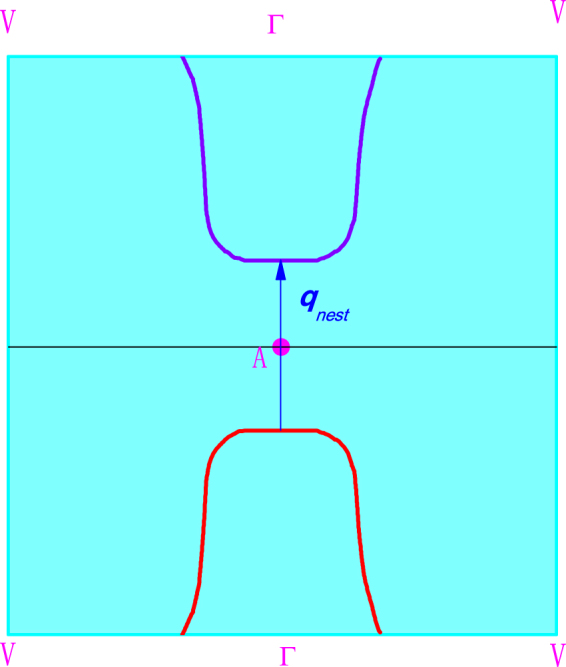
Electronic Structure. Calculated Fermi surface of Fe_3_Ga_4_ on a (010) plane through Γ with the proposed nesting illustrated. Here Γ = (0 0 0), *A* = (0 0 1/2) and *V* = (1/2 0 0). Red and violet are sheets of Fermi surface belonging to adjacent Brillouin zones.

To establish the itinerant nature of the magnetism, the electronic structure of Fe_3_Ga_4_ was calculated in the nonmagnetic and spin polarized phases employing the full potential linearized augmented plane wave method^29^. The resulting Fermi Surfaces were examined for possible nesting along the *c*-axis. Although we find no such nesting condition in the non-magnetic FS, the majority spin-band FS in the FM state contains a FS sheet with a substantial region that is flat and perpendicular to the c^*^ direction (see Fig. 4). Here, the nesting across the Brillouin zone boundary is demonstrated indicating the likely formation of density wave ordering. The nesting wavevector shown in this figure, *q*_*nest*_ = (0 0 0.276), is in very good agreement with the experimentally observed ISDW providing compelling evidence that the FM state is unstable to the formation of a density wave.

## Discussion

We have carried out an extensive neutron diffraction investigation of Fe_3_Ga_4_ solving, for the first time, the magnetic structure of what had been reported to be an AFM state finding, instead, an ISDW phase. The ground state is confirmed as a robust FM state and the re-emergence of this ferromagnetism is also confirmed for $360\le T\le 420$ K. Although the magnetic contribution to the neutron scattering signal is small and data too sparse above 360 K to make a convincing comparison between the low and high *T* FM states, our *χ*(*T*) and *M*(*H*,*T*) data display identical anisotropy as well as similar moment magnitudes and field dependencies indicating that these FM states are likely identical^20^. Furthermore, electronic structure calculations reveal a FS sheet in the majority band of the FM state that is unstable towards density wave formation with a nesting vector that matches the neutron data both in direction and magnitude. Interestingly, the ISDW state likely results from a FS instability in only one of the spin-polarized bands of Fe_3_Ga_4_, a mechanism that, thus far, has not been considered or identified in other materials. The details of how the transition between itinerant FM and ISDW states takes place are not yet clear since a residual magnetic moment may be expected for a simple FS reconstruction in conflict with our *M*(*H*)^20^ and neutron diffraction data.

These conclusions place Fe_3_Ga_4_ as a unique compound among the large number of FM materials reported in the literature such that identifying materials that are more than tangentially related is difficult. UCu_2_Si_2_ and CeRu_2_Al_2_B, where the Fermi surfaces are likely to be involved in the mechanism creating ISDW phases, are the closest comparisons that we have been able to find^17–19^. Extending the conversation to examples of FM materials with consequential FS nesting in their PM states broadens the number of comparisons slightly as this is only somewhat rare. Most of these are associated with competing orders and none, that we are aware of, report ISDW states resulting from nesting. Competing orders occur, for example, in SmNiC_2_ which has a charge density wave (CDW) phase that exists only above its Curie *T*^30^. In addition, nesting of the PM FS is common in the heavy rare earth elemental metals which tend to have FM ground states^10,11^. This nesting leads to transitions from PM into helimagnetic, rather than ISDW, states. More interesting is the idea that triplet superconductivity in FM UGe_2_ is related to coupled CDW-ISDW fluctuations that emerge with pressure within the FM phase^31^. Because the unusual transition between ISDW and FM states in Fe_3_Ga_4_ occurs just above room temperature, it has been suggested as a possible material for a magnetic tunneling junction device^32^. This suggestion takes advantage of the ability to drive a phase transition between the ISDW and spin-polarized phases with moderately sized magnetic fields and the ability to tune the phase transition temperature via pressure and disorder. Our findings here support the idea of Fe_3_Ga_4_ as a possible material for phase transition tunneling anisotropic magnetoresistance (PT-TAMR) devices since our simulations suggest a large difference in the electronic density of states at the Fermi level between the ISDW and FM (spin polarized) states.

We are left with two important unanswered questions about the mechanism we discovered for the transitions between FM and ISDW states in Fe_3_Ga_4_. The first is the character of the magnetism and its relationship to the conducting charge carriers. Somewhat in contrast to an itinerant magnetism in Fe_3_Ga_4_ are the large magnetic moments evident in *M*, simulation, and the neutron scattering cross-sections suggesting magnetic moments may be more localized to the Fe sites. This would indicate an important coupling mechanism between the charge carriers and the more localized electrons responsible for the magnetic moments. A similar cooperative mechanism has been proposed for GdSi where both RKKY and FS nesting are thought to play a role in creating an ISDW state^33^ and for the Fe-based superconducting families where this coupling is responsible for the various AFM structures^34–36^. The second related question is the cause of the FM ground state. We speculate that the re-emergence of FM ground state at low *T* may be a result of a loss of RKKY coupling with the establishment of partial energy gaps in the FS of the ISDW state, as has been suggested for the helimagnetic rare earth elemental metals, such as Dy and Tb^10,11,37,38^. In these materials an abrupt transition from FM-to-helimagnetic ordering results from superzone gaps in the FS of the incommensurate phase along with considerations of spin-orbit coupling^10^. As such, the incommensurate phase appears to be self limiting as the ordering creates a partial energy gap that removes carriers responsible for the RKKY coupling. Additionally, there is a complex and interesting variation of the magnetic wavevector and metamagnetic fields that is in many ways similar to what we observe in Fe_3_Ga_4_. Despite these remaining issues, our data and computational results indicate that Fe_3_Ga_4_ is the first material that has been discovered to evolve from a FM FS to an ISDW, a discovery that suggests that there is likely a rich and mostly unrecognized competition between magnetic states caused by FS instabilities of spin polarized bands.

## Methods

Single crystals of Fe_3_Ga_4_ with masses up to 100 mg were grown from high purity Fe powder and gallium pieces that were melted in a RF furnace prior to employing floating zone techniques. The resulting crystals were sealed in quartz tubes under vacuum and annealed at 550 °C for 48 hours.

The structure of the crystals was determined by single crystal X-ray diffraction using a Bruker D8 Quest Kappa single-crystal diffractometer (Mo *Kα IμS* microfocus source, $\lambda =0.71073$ Å) operating at 50 kV and 1 mA, a HELIOS optics monochromator, and a CMOS detector. These measurements confirmed a base-centered monoclinic structure having the space group *C*2/*m*^24,25^ for $100\le T\le 380$ K. Single crystal X-ray diffraction data were collected at 100, 300, 328, 350, and 380 K. Crystals were cut to an appropriate size and mounted onto a glass fiber using epoxy. The Bruker program SADABS was used to correct the collected data for absorption^39^. The intrinsic phasing method in SHELXT^40^ was used to obtain starting crystallographic models and SHELXL2014^41^ was used to refine atomic sites. The crystallographic parameters and atomic positions for Fe_3_Ga_4_ at 300 K are provided in Table S1 to serve as a representative of all the data collected. No evidence for an incommensurate scattering contribution at 100, 300, and 380 K that would indicate a possible strain wave associated with the incommensurate magnetic ordering was apparent in precession images.

We have also performed powder X-ray diffraction on ground crystals for $300\le T\le 670$ K and found no crystallographic changes beyond the expected thermal expansion and a small decrease in the angle *β*. This included temperature-dependent powder X-ray diffraction data collected at 300, 328, 350, 380, 450, and 670 K using a Bruker D8 Advance powder X-ray diffractometer operating at 40 kV and 30 mA with Cu K *α* radiation ($\lambda =1.54184$ Å) equipped with a temperature stage and a LYNXEYE XE detector. Data were collected in the 2*θ* range of 10–80° with a step size of 0.01°. Rietveld refinements were performed using the TOPAS5 software package. The scale factor, background (Chebyshev function with eight terms), and unit cell were independently refined.

Magnetic measurements carried out in a SQUID magnetometer revealed behavior indistinguishable from previously grown vapor transport crystals^20^. A single crystal with a mass of 24 mg was chosen for magnetic structure determination via neutron scattering using the HB-3A four-circle diffractometer at the High Flux Isotope Reactor of Oak Ridge National Laboratory employing a wavelength of 1.546 Å. The sample was placed in a closed-cycle refrigerator allowing exploration for $5\le T\le 450$ K. We established the FM order parameter by scanning the (111) and (201) Bragg reflections over the 5 to 450 K range. Wave-vector scans along the (2 0 *L*) direction with $0.4\le L\le 1.1$ were collected at a large number of temperatures to characterize the magnetic phase diagram. In addition, a large region of reciprocal space was scanned that included all of the accessible nuclear reflections as well as a large number of peaks associated with the ISDW ordering. Altogether, 94 peaks were used for a full refinement of the FM state at 5 K and 154 *q*-scans taken at 100 K were employed in determining the ISDW state. Refinement of both the nuclear and magnetic structures was performed using the FULLPROF Suite^42^. The correlation length for the magnetic scattering in the incommensurate spin density wave phase (*ξ*) was determined via a fit of the data by a Lorentzian function convoluted with the instrumental resolution.

Electrical transport measurements were made on rectangular shaped single crystals polished with emery paper. Four Epotek silver epoxy contacts with an average spacing between voltage probes of 0.3 mm were formed on the surface of the crystals to make electrical contact to thin Pt wires. Conductivity measurements were were performed at 19 Hz using standard lock-in techniques in a gas flow cryostat.

### Data availability

The authors declare that the main data supporting the findings of this study are available within this article and its Supplementary Information. Extra data are available from the corresponding author upon reasonable request. See author contributions for specific data sets.

## Electronic supplementary material

Supplementary Information
