# Supplementary material for: Spin density wave instability in a ferromagnet
Source: Sci Rep. 2018 Mar 27;8:5225. doi: 10.1038/s41598-018-23555-4 (PMC5869675; doi:10.1038/s41598-018-23555-4)
Supplement: Supplementary file 1 — Supplementary Information [file 41598_2018_23555_MOESM1_ESM.pdf]

# Supplementary Information for Spin density wave instability in a ferromagnet

Yan Wu[1], Zhenhua Ning[1], Huibo Cao[2], Guixin Cao[1], Katherine A. Benavides[3], S. Karna[1], Gregory T. McCandless[3], R. Jin[1], Julia Y. Chan[3], W. A. Shelton[4] & J. F. DiTusa[1]

[1]*Department of Physics and Astronomy, Louisiana State University, Baton Rouge, LA 70803*

[2]*Quantum Condensed Matter Division, Oak Ridge National Laboratory, Oak Ridge, TN 37831*

[3]*Department of Chemistry and Biochemistry, The University of Texas at Dallas, Richardson, TX 75080*

[4]*Department of Chemical Engineering, Louisiana State University, Baton Rouge, LA 70803.*

Here we present a summary of the crystal and magnetic structure of $Fe_3Ga_4$ determined from the X-ray and neutron diffraction experiments as well as a description of the refinement process to determine the magnetic structure in the intermediate temperature incommensurate phase.

$Fe_3Ga_4$ forms in a monoclinic ($C2/m$) crystal structure with no evidence of structural change associated with the magnetic phase transitions[1–5]. This structure is somewhat complex having 4 crystallographically unique Fe sites and 4 unique Ga sites within the unit cell (Fig. 1d and Table S1). The crystallographic parameters and atomic positions for $Fe_3Ga_4$ determined via single crystal X-ray diffraction at 300 K are provided in Table S1 to serve as a representative of all the data collected.

The results of the refinement of the neutron diffraction data are presented in Tables S2 and S3 which delineate both magnitude and orientation of the magnetic moments in the ferromagnetic low temperature state and the intermediate temperature spin density wave state. Previous neutron scattering investigations of the magnetic structure of $Fe_3Ga_4$ in polycrystalline samples were inconclusive[3,6,7].

The refinement process began with a representational analysis to determine the crystal structural symmetry allowed magnetic symmetry. The calculations were carried out using version 2K of the program SARA*h*-Representational Analysis[8]. They first involved the determination of the space group symmetry elements, $g$, that leave the propagation vector $k$ invariant. Those elements form the little group $G_{\mathbf{k}}$. The magnetic representation of a crystallographic site can then be decomposed in terms of the irreducible representations (IRs) of $G_{\mathbf{k}}$:

$$\Gamma_{Mag} = \sum_{\nu} n_{\nu} \Gamma_{\nu}^{\mu} \tag{1}$$

where $n_{\nu}$ is the number of times that $\Gamma_{\nu}$ of order $\mu$ appears in the magnetic representation $\Gamma_{Mag}$ for the chosen crystallographic site.

For $Fe_3Ga_4$, the crystal structure at temperatures above the phase transition is described by the space group C 2/m (#12:b1), which involves 2 centering operations and 4 symmetry operations. Only 2 operations leave the propagation $k$ invariant or transform it into an equivalent vector. These IRs are $\Gamma_1$ and $\Gamma_2$. The decomposition of the magnetic representation $\Gamma_{Mag}$ in terms of the two IRs of $G_{\mathbf{k}}$ for 7 Fe magnetic sites are examined. The basis vectors of Fe1-Fe5 sites only have contributions along the $b$-axis under $\Gamma_1$ and are confined in the $ac$-plane under $\Gamma_2$. All of the $a$, $b$, and $c$ components are allowed on the Fe6 and Fe7 sites in both IRs. Based on our neutron diffraction results, only the $\Gamma_2$ IR works for the system. The $\Gamma_2$ IR associated basis vectors were applied to the FullProf[9] refinement and the refinement results supports the incommensurate spin density wave (ISDW) state with $R_{factor}$ being 12.2A schematic of this magnetic state is shown in Fig. 1d and e and the magnetic moments are detailed in Table S3.

Since the incommensurate magnetic structures associated with ferromagnetic states are typically helimagnetic, we have carefully explored the possibility of the intermediate temperature state being a helical structure. A simulated annealing process was employed to search for the best fit model of a helical magnetic structure. We started from a model where the magnetic moments are confined to the ac plane, consistent with the symmetry analysis discussed above. The global ground state found under these conditions after 90 iterations retained a large $R_{factor}$ of 40.2. Fi-

nally, we searched for a helical magnetic structure without constraining the spin direction. A model magnetic structure was found with magnetic moments rotating in the $ab$-plane having an $R_{factor}$ of 13.2, comparable to that of the ISDW model. However, this state is not compatible with either the magnetic representation symmetry or the anisotropy we measure in the bulk magnetization (see Fig. 3a) so that it is not considered an acceptable magnetic structure for $Fe_3Ga_4$. Thus, the refinement procedure leads to the conclusion that the intermediate temperature state of $Fe_3Ga_4$ is an ISDW state which is consistent with our bulk measurements.


1. Mendez, J. H. *et al.* Competing magnetic states, disorder, and the magnetic character of $Fe_3Ga_4$. *Phys. Rev. B* **91**, 144409 (2015).

2. Duijn, H. G. M. *et al.* Pressure dependence of the ferromagnetic to antiferromagnetic transition in $Fe_3(Ga_{1-x}Al_x)_4$ with $x = 0.0$ and 0.1. *J. Appl. Phys.* **85**, 4738–4740 (1999).

3. Duijn, H. *Magnetotransport and magnetocaloric effects in intermetallic compounds*. Ph.D thesis, Universiteit van Amsterdam (2000).

4. Philippe, M., Malaman, B. & Roques, B. Preparation and study of intermetalliciron-gallium compounds in single-crystal state. *Comptes Rendus Hebdomadaires Des Seances De L Academie Des Sciences Serie C* **278**, 1093–1095 (1974).

5. Philippe, M., Malaman, B., Roques, B., Courtois, A. & Protas, J. Crystal-structures of phases $Fe_3Ga_4$ and $Cr_3Ga_4$. *Acta Crystallographica Section B-Structural Science* **31**, 477–482 (1975).

6. Al-Kanani, H. J. & Booth, J. G. High field transitions in $(Fe,T)_3Ga_4$ alloys. *Physica B* **211**, 90–92 (1995).

7. Al-Kanani, H. J., Booth, J. G. & Ko, K. Y. Magnetic phase transitions in $(Fe_{1-x}T_x)_3Ga_4$ alloys. *J. Appl. Phys.* **87**, 4879–4881 (2000).

8. Willis, A. S. A new protocol for the determination of magnetic structures using simulated annealing and representational analysis (SARAh). *Physica B* **276**, 680–681 (2000). Program available from www.ccp14.ac.uk.

9. Rodriguez-Carvajal, J. Recent advances in magnetic structure determination by neutron powder diffraction. *Physica B* **192**, 55 (1993). For a more recent version see Rodriguez-Carvajal, J. Recent Developments of the Program FULLPROF, in Commission on Powder Diffraction (IUCr) Newsletter , **26**, 12-19 (2001).

| **Label** | **Wyckoff Pos.** | **x** | **y** | **z** | $U_{eq}(\text{Å}^2)^a$ |
|---|---|---|---|---|---|
| Fe1 | 2a | 0 | 0 | 0 | 0.00314(18) |
| Fe2 | 4i | 0.49454(7) | 0 | 0.31074(9) | 0.00373(14) |
| Fe3 | 4i | 0.22477(7) | 0 | 0.63799(9) | 0.00320(14) |
| Fe4 | 8j | 0.13570(5) | 0.20036(6) | -0.15173(6) | 0.00356(12) |
| Ga1 | 4i | 0.26982(6) | 0 | 0.08459(7) | 0.00667(13) |
| Ga2 | 4i | 0.04142(6) | 0 | 0.34795(7) | 0.00608(13) |
| Ga3 | 8j | 0.60842(4) | 0.20288(5) | 0.15120(5) | 0.00586(11) |
| Ga4 | 8j | 0.35199(4) | 0.18656(5) | 0.44954(5) | 0.00703(11) |

Table S1: **Atomic Positions of $Fe_3Ga_4$ at 300 K determined from single crystal X-ray diffraction**: Space Group $C2/m$, $a$ = 10.0949(16) Å, b = 7.6603(16) Å, c = 7.8703(14) Å, $\beta$ = 106.286(2)°

| **Atom** | **Atomic Coordinates** | | | **Moment($\mu_B$)** |
|---|---|---|---|---|
| | **a** | **b** | **c** | $m_c$ |
| Fe1 | 0 | 0 | 0 | 1.54(28) |
| Fe2 | 0.4935(6) | 0 | 0.3096(7) | 1.20(22) |
| Fe3 | 0.2240(7) | 0 | 0.6376(8) | 1.24(21) |
| Fe4 | 0.1352(5) | 0.2019(5) | 0.8481(6) | 1.49(12) |
| Average Moment | | | | 1.37(22) |

Table S2: **Magnetic structure data for the low temperature ferromagnetic state**: magnetic moments determined from the refinement of the neutron scattering data at 5 K for the four in-equivalent crystallographic Fe sites. Moments were constrained to lie along the c-axis.

| Atom | Atomic Coord. | | | Magnetic Moments | | | $m_{mean}(\mu_B)$ | | | | $m_{max}(\mu_B)$ | | | |
|---|---|---|---|---|---|---|---|---|---|---|---|---|---|---|
| Site | a | b | c | a | b | c | a | b | c | $\lvert m \rvert$ | a | b | c | $\lvert m \rvert$ |
| 1 | 0 | 0 | 0 | -2.31(6) | 0 | 0.58(2) | 1.47(4) | 0 | 0.37(1) | 1.51(4) | 2.31(6) | 0 | 0.58(1) | 2.38(6) |
| 2 | 0.4935(6) | 0 | 0.3096(7) | -1.55(4) | 0 | 0.38(1) | 1.02(2) | 0 | 0.25(1) | 1.05(4) | 1.61(4) | 0 | 0.40(1) | 1.65(4) |
| 3 | 0.5065(6) | 0 | 0.6904(7) | -0.96(3) | 0 | 0.24(1) | 1.02(2) | 0 | 0.25(1) | 1.05(4) | 1.61(4) | 0 | 0.40(1) | 1.65(4) |
| 4 | 0.2240(7) | 0 | 0.6376(8) | -0.95(3) | 0 | 0.24(2) | 1.16(3) | 0 | 0.29(1) | 1.20(5) | 1.83(4) | 0 | 0.46(1) | 1.88(5) |
| 5 | 0.7760(7) | 0 | 0.3624(8) | -1.79(5) | 0 | 0.45(2) | 1.16(3) | 0 | 0.29(1) | 1.20(5) | 1.83(4) | 0 | 0.46(1) | 1.88(5) |
| 6(2) | 0.1352(5) | 0.2019(5) | 0.8481(6) | -0.22(1) | 0.06(1) | -0.05(1) | 1.32(2) | 0.37(4) | 0.33(1) | 1.41(8) | 2.08(3) | 0.58(6) | 0.52(1) | 2.22(8) |
| 7(2) | 0.8648(5) | 0.2019(5) | 0.1519(6) | -2.00(3) | -0.56(7) | 0.50(1) | 1.32(2) | 0.37(4) | 0.33(1) | 1.41(8) | 2.08(3) | 0.58(6) | 0.52(1) | 2.22(8) |
| $m_{mean}$ | | | | 0 | 0 | 0 | 1.24(3) | 0.16(2) | 0.31(1) | 1.30(4) | - | - | - | - |
| $m_{max}$ | | | | - | - | - | - | - | - | - | 2.31(6) | 0.58(7) | 0.58(2) | 2.38(8) |

Table S3: **Details of the magnetic structure of the spin density wave state**: Magnetic structural data for the spin density wave state at 100 K from refinement of our neutron scattering data. Data presented in this table include atomic coordinates determined from the structure refinement at $T$= 5 K, the magnetic moment in a representative single primitive cell labeled 'magnetic moments', the mean magnetic moment, $m_{mean}$, averaged over a large number of unit cells, and the amplitude, $m_{max}$, for each of the different crystallographic directions. The symmetry reduction in the incommensurate spin density wave state results in in-equivalent magnetic moments on 7 different Fe sites (6 and 7 have 2 fold symmetry).
